# MDL-1, a growth- and tumor-suppressor, slows aging and prevents germline hyperplasia and hypertrophy in *C. elegans*

**DOI:** 10.18632/aging.100638

**Published:** 2014-02-16

**Authors:** Michèle Riesen, Inna Feyst, Nattaphong Rattanavirotkul, Marina Ezcurra, Jennifer M.A. Tullet, Irene Papatheodorou, Matthias Ziehm, Catherine Au, Ann F. Gilliat, Josephine Hellberg, Janet M. Thornton, David Gems

**Affiliations:** ^1^ Institute of Healthy Ageing, and Research Department of Genetics, Evolution and Environment, University College London, London, United Kingdom; ^2^ European Molecular Biology Laboratory, European Bioinformatics Institute, Wellcome Trust Genome Campus, Hinxton, Cambridge, United Kingdom

**Keywords:** aging, C. elegans, FoxO, germline, hyperplasia, hypertrophy, Mad transcription factor

## Abstract

In *C. elegans*, increased lifespan in *daf-2* insulin/IGF-1 receptor mutants is accompanied by up-regulation of the MDL-1 Mad basic helix-loop-helix leucine zipper transcription factor. Here we describe the role of *mdl-1* in *C. elegans* germline proliferation and aging. The deletion allele *mdl-1(tm311)* shortened lifespan, and did so significantly more so in long-lived *daf-2* mutants implying that *mdl-1(+)* contributes to effects of *daf-2* on lifespan. *mdl-1* mutant hermaphrodites also lay increased numbers of unfertilized oocytes. During aging, unfertilized oocytes in the uterus develop into tumors, whose development was accelerated by *mdl-1(tm311)*. Opposite phenotypes were seen in *daf-2* mutants, i.e. *mdl-1* and *daf-2* mutant germlines are hyperplastic and hypoplastic, respectively. Thus, MDL-1, like its mammalian orthologs, is an inhibitor of cell proliferation and growth that slows progression of an age-related pathology in *C. elegans* (uterine tumors). In addition, intestine-limited rescue of *mdl-1* increased lifespan but not to wild type levels. Thus, *mdl-1* likely acts both in the intestine and the germline to influence age-related mortality.

## INTRODUCTION

In most animals, advancing age is accompanied by the deteriorative process of aging (senescence). Aging is the main cause of severe illness and death in humans, but the proximate biological mechanisms that cause it have proved difficult to identify. One approach to understand aging is to study simple model organisms [1], such as the nematode *Caenorhabditis elegans* which is particularly suitable for this purpose given e.g. its sequenced genome and very short lifespan (2-3 weeks). The identification from the 1980s onwards of many *C. elegans* mutants with altered aging rate [1] led to optimism that discovery of gene products of aging control genes would reveal the mechanisms of aging in this organism. Yet although many signaling pathways and processes affecting aging rate have been identified, the nature of aging itself has remained obscure. For example, mutation of the *daf-2* insulin/IGF-1 receptor gene can more than double adult lifespan [2]. This increase requires the presence of the DAF-16 FoxO transcription factor [2-4], suggesting that transcriptional targets of DAF-16 encode proximal biochemical determinants of aging. But these target genes have proved to be very numerous [5, 6], 2,274 by one estimate [7], complicating the search for DAF-16 target genes that control aging. Understanding DAF-16/FoxO action is important, particularly because the role of insulin/IGF-1 signaling and FoxO in the control of aging shows evolutionary conservation, e.g. in the fruitfly Drosophila [8], and perhaps even in humans, where age changes in allele frequency e.g. of the IGF-1 receptor and FoxO3A genes have been detected [1].

**Figure 1:**
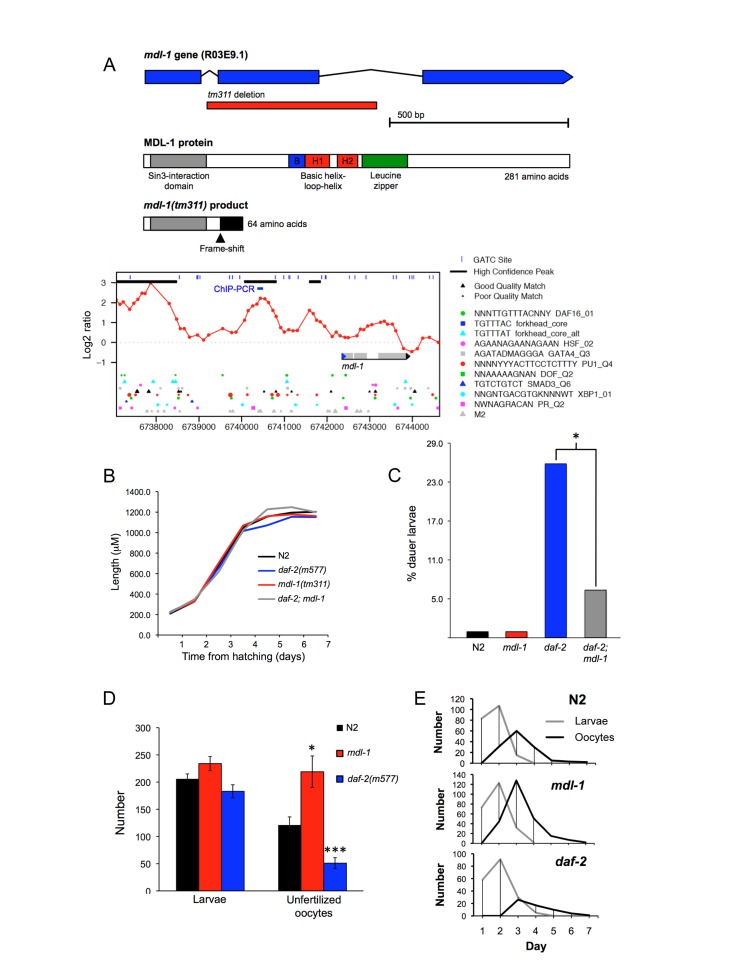
Phenotypic analysis of *mdl-1(tm311)* mutant (**A**) *mdl-1* gene and protein description, including *tm311* deletion and effects on protein, DAF-16 binding sites (chromatin profile [DamID] data and DBEs) and site of ChIP analysis. For chromatin profile, y axis represents log2 ratio of DAF-16 binding relative to control, and peaks correspond to potential DAF-16 binding sites [9]. (**B-F**). Phenotypic effects of *mdl-1(0)*. (**B**) Little effect on larval and adult growth. Samples sizes ranged from 23-39. (**C**) *mdl-1(0)* slightly reduces *daf-2(m577)* Daf-c, measured at 22.9°C. * 0.01 < *p* < 0.05 (Student's *t* test). 4 trials conducted, in which *mdl-1(0)* reduced dauer formation in 3. (**D, E**) Effect of mdl-1 and daf-2 on fertility. Number of broods scored: N2, 19; *daf-2*, 20; *mdl-1*, 17. (**D**) Mean total progeny and unfertilized oocytes. * 0.01 < *p* < 0.05, *** *p* < 0.001 (Student's t test). (**E**) Mean daily progeny and unfertilized oocyte numbers.

One approach to understand DAF-16 action is to map the gene regulatory network in which it acts. Previously we used a genome-wide approach to identify genes to which DAF-16 both binds and causes a change in gene expression [9]. This identified a mere 65 high confidence DAF-16 direct targets, which were enriched for genes encoding proteins involved in signaling and gene regulation, and transcription factors. Among the latter class was *mdl-1* (Mad-like 1), which encodes a basic helix-loop-helix (bHLH) TF homologous to mammalian Mad transcription factors [10] (Figure 1A). In mammals, Mad TFs act as heterodimers with Max bHLH TFs. Mad competes with Myc bHLH TFs to dimerize with Max, and bind to target genes containing E-box sequences (5'-CANNTG-3') [11]. Myc/Max dimers mainly activate gene expression, and are a major activator of cell proliferation of growth. By contrast, Mad/Max dimers mainly inhibit gene expression, antagonizing Myc/Max, and suppressing cell division and growth [11]. Inhibition of gene expression by Mad/Max is facilitated by recruitment of the Sin3 histone deacetylase (HDAC) corepressor complex. Myc TFs are potent oncogenes, while Mad TFs show some properties of tumor suppressors [11].

Our attention was drawn to *mdl-1* for several reasons. First, many genes that promote growth also promote aging [12]. Thus, growth suppressors activated by DAF-16 are candidates for downstream effectors slowing aging, and MDL-1, as a Mad TF, is a potential growth suppressor and, in fact, can suppress activated cMyc/Ras-induced cell transformation in mammalian cells [10]. Second, four mammalian Mad TFs, *mad1, mxi1, mad3* and *mad4*, are up-regulated by FoxO3a in a human colorectal adenocarcinoma cell line [13]. Thus, regulatory interactions between FoxO and Mad show at least some evolutionarily conservation between nematodes and mammals. Consistent with this, in *C. elegans mdl-1* is an activator of intestinal expression of *ftn-1* (H ferritin, an iron storage protein) [14], while in mammals, Myc can repress H ferritin expression, which contributes to cell proliferation [15].

*C. elegans* possesses several Max-like (*mxl*) genes, including *mxl-1* which can form heterodimers with MDL-1 but, surprisingly, lacks Myc [10, 16, 17]. Previous RNAi screens have not detected major effects of expression knockdown of *mdl-1* or *mxl-1* (Wormbase.org). However, *mdl-1* exerts some influence upon the germline, as follows. Loss of *daf-2* inhibits lethal, *gld-1*-induced distal germline tumors via decreased cell division and increased DAF-16/p53-dependent apoptosis [18], and *mdl-1* is a mediator of this inhibition [19]. Moreover, RNAi of *mdl-1* can reduce *daf-2* mutant longevity, but has little effect on lifespan in *daf-2* (+)worms [6].

In this study, we explore the possible role of *mdl-1* as a downstream effector of DAF-16 in the control of aging. In particular, we detail the phenotypic effects of mutation of *mdl-1*. We report that *mdl-1* acts as a repressor of germline hyperplasia and hypertrophy which otherwise contributes to age-related pathology in the germline.

## RESULTS

### *mdl-1(tm311)* increases production of unfertilized oocytes

To investigate *mdl-1* gene function, we studied the *mdl-1(tm311)* mutant allele, which contains a 471 bp base pair deletion that removes exon 2 of the gene (Figure 1A). This results in a frame shift after 51/281 amino acid residues and loss of the entire bHLH domain, implying that this is a null allele. The mutation was first backcrossed 6x into the *Caenorhabditis* Genetics Center wild type male stock to remove possible second site mutations, and ensure a wild type background [20]. Previous work on *mdl-1* and the function of Mad TFs in mammals led to several expectations about the possible effects of *mdl-1(0)*. First, since it is a DAF-16-activated gene, it might suppress *daf-2* mutant traits, e.g. constitutive dauer larva formation (Daf-c), stress resistance and increased longevity (Age). Second, since Mad TFs inhibit cell division and growth, *mdl-1(0)* might increase either somatic growth or germline proliferation.

We first examined effects of *mdl-1(0)* on somatic development and growth in wild type and *daf-2* mutant backgrounds. *mdl-1(0)* had no detectable effect on larval or adult growth (Figure 1B), but caused a slight reduction in constitutive dauer formation in *daf-2(m577)* mutants (Figure 1C). Next we probed the effects of *mdl-1* on the germline, first by looking at levels of fertility. The number of progeny produced by self-fertilized hermaphrodites was not affected by *mdl-1(0)*, either in terms of overall brood size or reproductive schedule (Figure 1D,E). As self sperm becomes depleted, N2 hermaphrodites start laying unfertilized oocytes [21]. Notably, *mdl-1(0)* caused a marked increase in the number of unfertilized oocytes laid, from 121 ± 15 to 219 ± 30, an 81% increase (Figure 1D). *mdl-1*(RNAi) applied to RNAi-sensitive *rrf-3(pk1426)* mutants also increased unfertilized oocyte number (data not shown).

In *daf-2(m577)* mutants, progeny number was also not different to N2, but the number of unfertilized oocytes laid was significantly reduced (Figure 1D,E), consistent with previous findings [22]. For convenience, to describe this mutant phenotype we introduce the term Uno (abnormal in unfertilized oocyte production), and Uno-o, to describe mutants that are unfertilized oocyte over-producers (e.g. *mdl-1*), and Uno-d, to describe mutants that are unfertilized oocyte deficient (e.g. *daf-2*).

### *mdl-1(tm311)* causes germline hyperplasia and hypertrophy

**Figure 2:**
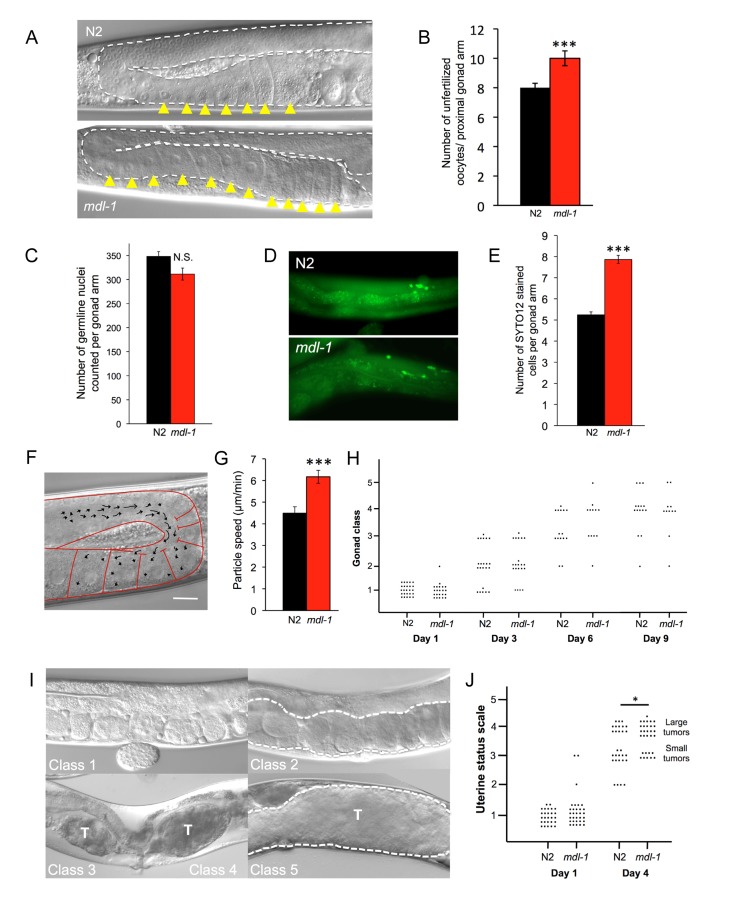
*mdl-1* causes hyperplasia and hypertrophy **(A, B)** *mdl-1(0)* causes oocyte stacking in 1 day old worms. **(A)** Nomarski images. **(B)** Quantitation of stacking. Sample sizes: N2, 23; *mdl-1*, 17. *** *p* < 0.001 (Student's *t* test). **(C)** No effect of *mdl-1* on number of germline nuclei. *p* > 0.05 (Student's *t* test). **(D, E)** *mdl-1* increases levels of germline apoptosis. **(D)** Epifluorescence images of SYTO12 stained cells in young adult hermaphrodite germline. **(E)** Quantitated data. Number of gonads scored: N2, 153; *mdl-1*, 132. *** *p* < 0.001 (Student's *t* test). **(F, G)***mdl-1* increases cytoplasmic streaming in the proximal gonad. **(F)** Single image obtained from a time-lapse recording. Arrows represent DIC-particle tracks. DIC-particles were tracked over a period of 1 minute. Scale bar: 20 μm. **(G)** Cytoplasmic streaming rate (mean particle speed ± standard error). 30 particle speed measurements performed for each genotype. Number of worms examined: N2, 4; *mdl-1*, 3. *** *p* < 0.001 (Student's *t* test). **(H)** Absence of effect of *mdl-1(0)* on gonad disintegration (25°C). *p* > 0.05 for all comparisons of N2 *vs. mdl-1* of the same age (Wilcoxon Mann test). **(I)** Uterine status scale for quantitation of uterine tumor formation rate (5 classes). Class 1, normal uterus containing eggs (day 1 adult). Class 2, slightly abnormal uterine contents, but no tumor visible. Class 3, small tumor. Class 4, medium sized tumor. Class 5, large tumor, filling body cavity and squashing the intestine. Dotted line, outline of uterus. T, tumor. **(J)** *mdl-1(0)* increases uterine tumor formation (25°C), data summed from 3 trials. * 0.01 < *p* < 0.05 (Wilcoxon Mann test).

The *mdl-1* Uno-o phenotype suggests increased cell production in the germline distal to the spermatheca. To test this we compared proximal gonad contents in wild type and *mdl-1* animals on day 1 of adulthood. This revealed increased oocyte density, or stacking [23, 24], in *mdl-1* (Figure 2A,B), implying increased oocyte synthesis. This in turn suggests increased germ cell proliferation in the distal gonad. To probe this, we examined germ cell number by staining nuclei with the fluorescent DNA-binding dye 4',6-diamidino-2-phenylindole (DAPI), but no effect of *mdl-1* was detected (Figure 2C). However, an increase in the overall rate of germline cell turnover in *mdl-1* mutants could leave cell number unaffected.

If the distal proliferative zone is the source of germ cell nuclei, then the major sink is germline apoptosis. At least 50% [25] and as many as 97% [26] of germ cells undergo p53-independent, “physiological” apoptosis, their cytoplasm supplying expanding oocytes near the gonad bend. Using the SYTO 12 dye to detect apoptotic cell corpses, we found that *mdl-1* mutants showed a significant increase in apoptotic cell number in the germline in 3 out of 4 trials (Figure 2D, E).

The transfer of cytoplasm released by germ cells to nascent oocytes occurs by a process of cytoplasmic streaming (Figure 2F) [27]. We examined the effect of *mdl-1(0)* on the rate of cytoplasmic streaming in the mid-late pachytene region of the distal gonad, on day 1 of adulthood. Cytoplasmic streaming rate in *mdl-1* worms was significantly greater than in wild type (Figure 2G). Taken together, these results suggest that an increase in production of germ cells is matched by an increase in apoptosis, resulting in little change in overall germ cell number in the distal arm. Overall, this suggests that the increase in oocyte production is driven by a hyperplastic state in the distal gonad.

Next we studied the effect of *mdl-1* on pathologies of aging in the germline. The aging hermaphrodite gonad undergoes dramatic pathological changes. The distal gonad shrivels and eventually disintegrates [28, 29], while in the uterus large, amorphous masses (tumors) with very high DNA content develop [24, 29-31]. These tumors form from unfertilized oocytes which undergo multiple rounds of endoreduplication, and can grow to fill the entire body cavity in the mid-body. Continued germline apoptosis in late life contributes to gonad disintegration, and increased apoptosis rate is sufficient to increase gonad disintegration rate (Y. de la Guardia and D. Gems, unpublished). However, despite their increased apoptosis rate (Figure 2D,E) gonad disintegration rate was not detectably altered in *mdl-1* mutants (Figure 2H).

Casual observation of *mdl-1* hermaphrodites under Nomarski microscopy suggested an increase in uterine tumors in these mutants. To verify this, we used a semi-quantitative approach [28] with a uterine status scale. According to the appearance of the uterus, worms were scored from 1 (healthy, no tumors) to 5 (large tumors) (Figure 2I) (see Materials and Methods). Using this scale to compare N2 and *mdl-1* mutants confirmed that uterine tumors grow significantly faster in *mdl-1* worms (Figure 2J).

### *mdl-1* does not mediate effects of *daf-2* on germline proliferative status

**Figure 3:**
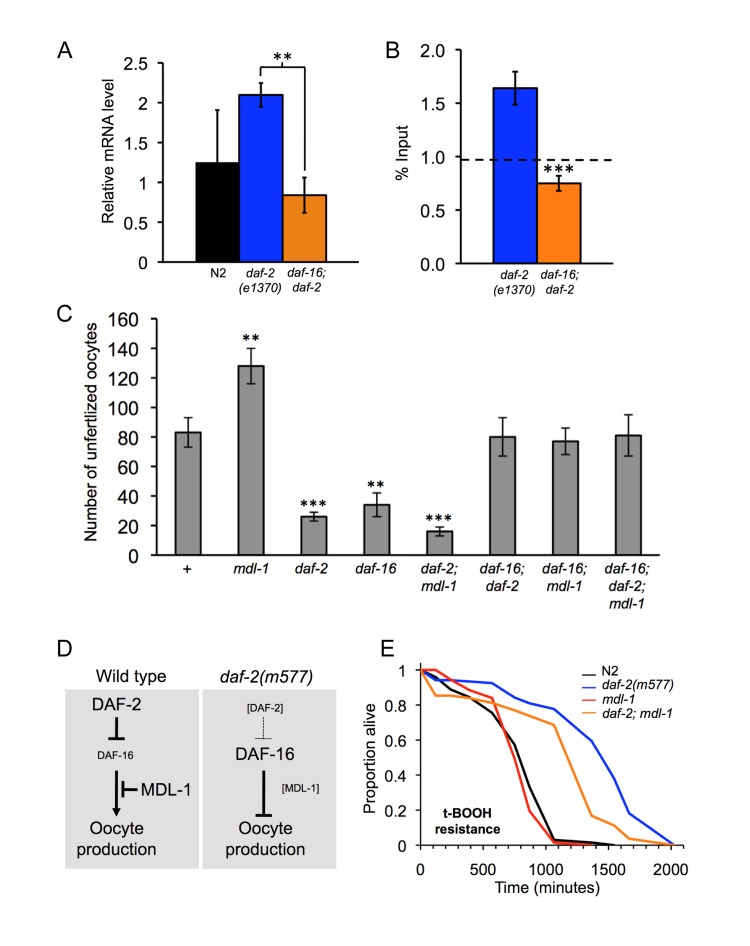
Distinct epistatic relationships between *daf-2* and *mdl-1* in hyperplasia and hypertrophy **(A, B)**. *mdl-1* is a direct transcriptional target of DAF-16. **(A)**. *mdl-1* mRNA levels are increased in *daf-2* relative to *daf-2; daf-16* (Q-PCR data). ** 0.001 < *p* < 0.01. **(B)** DAF-16 binds to the *mdl-1* promoter (ChIP-PCR data). One experiment is shown which contained 3 immunoprecipitation replicates from the same chromatin preparation (error bars show the standard deviation between them). The dotted line shows the average inputs from 3 genes/genomic regions that do not show enrichment for DAF-16 binding in *daf-2 vs daf-16; daf-2* in this particular trial, i.e. it reflects background DAF-16 binding levels. Significant DAF-16 binding was detected one of two additional trials. The position of the DAF-16 binding site detected is shown in Figure 1A. **(C)***mdl-1*, *daf-2* and *daf-16* epistasis analysis with respect to unfertilized oocytes production (Uno). Total unfertilized oocyte production per worm was measured at 25°C. Means of 12 broods assessed; error bars, standard error. ** 0.001 < *p* < 0.01; *** *p* < 0.001 (Student's *t* test). **(D)** Model for interactions between DAF-2, DAF-16 and MDL-1, deduced from interactions between mutations. DAF-16 promotes oocyte production in *daf-2*(+) worms, but inhibits it in *daf-2(m577)* worms. MDL-1 acts via DAF-16 to inhibit oocyte formation in *daf-2*(+) worms, but does not influence oocyte production in *daf-2(m577)* worms. **(E)** Resistance to 7.5 mM *tert*-butylhydroperoxide (*t*-BOOH). Sample sizes (censored values): N2, 67 [7]; *daf-2(m577)*, 61 [8]; *mdl-1,* 67 [8]; *daf-2; mdl-1,* 70 [12]. Probability of being the same: N2 *vs.mdl-1*, *p* = 0.24; *daf-2vs.daf-2; mdl-1*, *p* < 0.001 (log rank test).

We next investigated whether MDL-1, like DAF-16, is an effector of *daf-2* mutant phenotypes. We first verified that DAF-16 acts directly on *mdl-1* to increase its expression, as predicted by mRNA and chromatin profiling studies [9]. Quantitative RT-PCR confirmed that *mdl-1* mRNA levels are higher in *daf-2* than in *daf-16; daf-2* strains (Figure 3A). Chromatin immunoprecipitation and PCR (ChIP-PCR) confirmed that DAF-16 binds to the *mdl-1* promoter (Figure 3B). This implies that *mdl-1* expression is activated by DAF-16 binding to its promoter.

*mdl-1* mutants are Uno-o while *daf-2* mutants are Uno-d (Figure 1D,E) [22], and DAF-16 activates *mdl-1* expression (Figure 3A,B) [9]. This could imply that increased *mdl-1* activity in *daf-2* mutants reduces oocyte production. To test this we asked whether *mdl-1(tm311)* would suppress *daf-2* Uno-d, but it did not. Instead, *daf-2(m577);mdl-1* worms were Uno-d (Figure 3C), i.e. *daf-2* is epistatic to *mdl-1*. *daf-2(m577)* also suppressed *mdl-1* effects on oocyte stacking and uterine tumor formation (data not shown). These results negate our hypothesis that *daf-2* Uno-d is caused by *mdl-1* over-activity. A different model was suggested by additional epistasis data as follows. In a *daf-2*(+) background, *daf-16* suppressed *mdl-1* Uno-o, consistent with the observation that *daf-16* over-expression in a *daf-2*(+) background can cause germline hyperplasia [32]. Thus, DAF-16 promotes oocyte production in a *daf-2*(+) background but inhibits it in a *daf-2(m577)* background (Figure 3D). Moreover, mutation of *daf-16* in a *daf-2; mdl-1* mutant did not restore MDL-1 Uno-o (Figure 3C). This suggests that MDL-1 suppresses the effect of DAF-16 on oocyte production in *daf-2*(+) worms, but plays no role in *daf-2* mutants (Figure 3D).

*daf-2* mutants exhibit various forms of stress resistance, including oxidative stress resistance (Oxr) [33]. We tested whether MDL-1 contributes to *daf-2* Oxr, specifically to *tert*-butylhydroperoxide (*t*-BOOH). In a wild-type background, *mdl-1(0)* did not affect Oxr, while *daf-2(m577)* markedly increased Oxr (Figure 3E). Notably, in a *daf-2(m577)* background, *mdl-1(0)* significantly decreased Oxr. This implies that MDL-1 contributes to *daf-2* Oxr.

### MDL-1 contributes to *daf-2* mutant longevity by reducing baseline hazard

**Figure 4:**
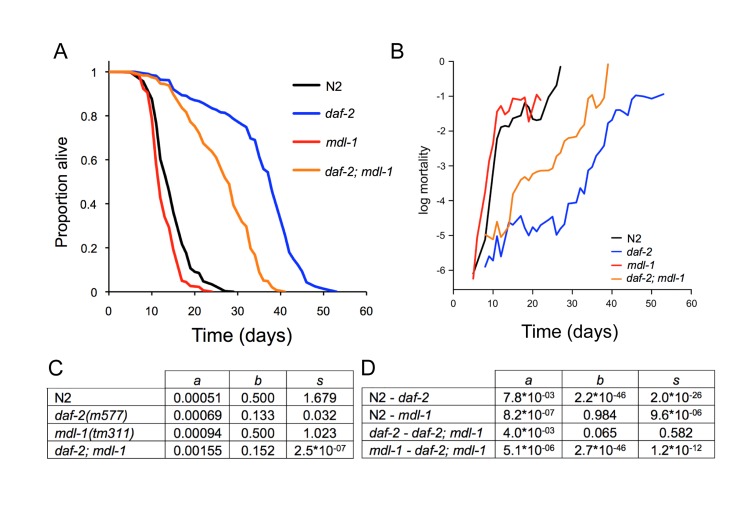
Effects of *mdl-1* on aging **(A)** Effects of *mdl-1(tm311)* on lifespan (for statistics, see Table S1, combined data). **(B)** Effect of *mdl-1* on age-specific mortality profiles. **(C, D)** Mortality analysis using logistic model. **(C)** Estimated values of logistic model parameters. **(D)** Probability, *p*, of parameters in compared genotypes being the same, holding other parameters constant.

Next, we examined the effect of *mdl-1* on aging. First we compared effects of *mdl-1*(RNAi) on lifespan in *rrf-3* and *rrf-3; daf-2(e1368)* strains (25°C), and detected a reduction in lifespan only in the latter strain (data not shown), consistent with previous observations [6]. We then assessed the effect of *mdl-1(tm311)* on lifespan in wild-type or *daf-2(m577)* mutant backgrounds. *mdl-1* decreased lifespan in both wild-type and *daf-2* backgrounds, but the decrease was proportionally greater in the latter (Figure 4A, Table S1). This corresponded to a significantly greater *mdl-1*-induced increase in mortality in a *daf-2* background (4.5-fold vs 1.8-fold; significant interaction term, *p* < 10^−15^, Cox proportional hazard analysis), suggesting that *mdl-1* activity contributes to the *daf-2* longevity increase.

To further characterize the effect of *mdl-1* on aging, we examined its effect on the pattern of age-specific mortality in wild-type and *daf-2(m577)* backgrounds.

Aging animal populations typically show exponential increases in mortality rate, and in *C. elegans* this occurs in two stages, with an initial faster exponential increase and a subsequent slower exponential increase [34, 35]. We fitted mortality data to a logistic model, which contains 3 components: a baseline hazard (initial mortality rate, parameter *a*), a mortality increase rate (parameter *b*) and a late-life mortality deceleration (parameter *s*).

In this analysis we wanted to probe whether *mdl-1(tm311)* shortens lifespan by accelerating aging or whether it could act by a life-shortening effect unrelated to aging. An effect of *mdl-1* on parameters *b* and *s* would imply an effect on aging, whereas an effect on parameter *a* could imply a non-aging related deleterious effect. In fact, *mdl-1* increased baseline hazard (*a*) without affecting the mortality increase rate (*b*) (Figure 4B-D). By contrast, relative to wild type, *daf-2(m577)* markedly decreased parameters *b* and *s*, while also slightly increasing parameter *a*. Reducing insulin/IGF-1 signaling has long been known to slow the age-related mortality rate increase [36]. In a *daf-2* background, *mdl-1(0)* again increased baseline hazard, and had no significant effect on parameters *b* and *s* (*p* > 0.05) (Figure 4C,D). In summary, this analysis confirms that *daf-2* increases lifespan by slowing demographic aging, while *mdl-1* shortens lifespan mainly by increasing baseline hazard. That *mdl-1* shortens lifespan more in a *daf-2* background could imply that MDL-1 contributes to *daf-2* longevity by reducing baseline hazard (see Discussion).

### Uterine tumors do not limit lifespan

**Figure 5:**
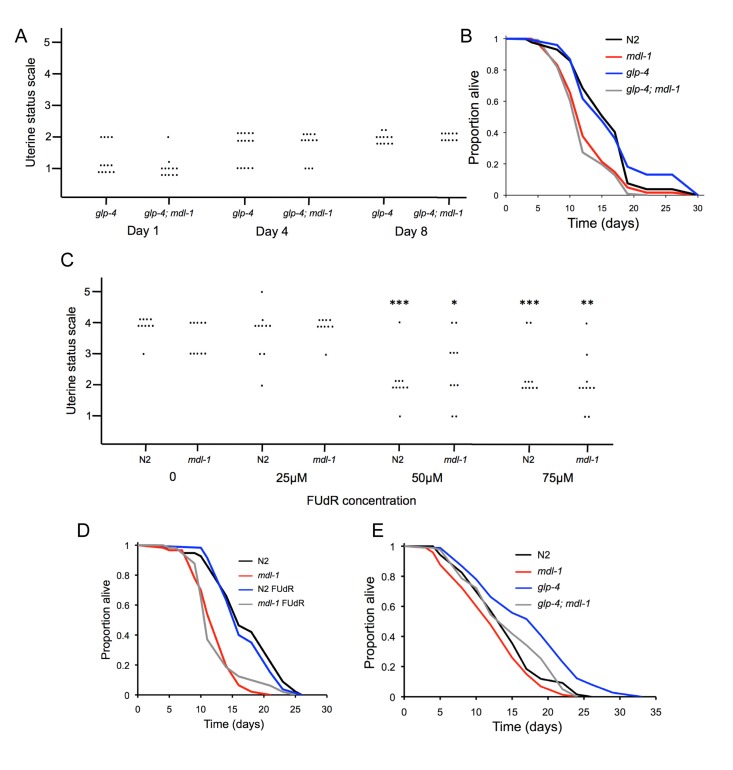
No effect of uterine tumors on lifespan **(A)** Uterine tumors are not seen in *glp-4(bn2)* mutants (raised at 15°C to L4 then shifted to 25°C). Uterine classes 2-5 indicate presence of tumors. Each dot corresponds to a uterine status measurement. **(B)** *glp-4* does not suppress *mdl-1* effects on lifespan (for statistics, see Table S2, trial 1). **(C)** FUdR suppresses formation of uterine tumors at 50μM or greater. Stars represent a significant difference to worms of the same genotype in the absence of FUdR. No significant difference in tumor levels between N2 and *mdl-1* were detected at this age at any FUdR concentration. * 0.01 < *p* < 0.05; ** 0.001 < *p* < 0.01; *** *p* < 0.001 (Wilcoxon Mann test). **(D)** 50μM FUdR does not suppress *mdl-1* shortevity or increase N2 lifespan (for statistics, see Table S2, trial 1). **(E)** Effect of *mdl-1* on *glp-4* longevity (for statistics, see Table S2, trial 2).

*mdl-1(0)* accelerates formation of uterine tumors, which frequently grow very large, filling the body cavity in the mid-body region and squashing the intestine [31]. One possibility is that uterine tumors can contribute to mortality and that increased tumor formation in *mdl-1(0)* mutants causes a shortened lifespan. To test this, we examined the effect of *mdl-1* on lifespan in the absence of uterine tumors. *glp-4(bn2)* mutants have a temperature-sensitive germline proliferation defect; if raised at 15°C to L4 and then switched to 25°C, oocyte production is blocked but longevity is not increased [7]. We first confirmed that *glp-4* blocks formation of uterine tumors (Figure 5A). We then compared lifespan in *glp-4*, *mdl-1* and *glp-4; mdl-1* worms. *glp-4* worms were normal-lived, but both *mdl-1* strains were similarly short lived (Figure 5B; Table S2).

We also blocked uterine tumor formation using the inhibitor of DNA replication 5-fluoro-deoxyuridine (FUdR), which is also commonly used to treat colorectal cancer. Application of FUdR at low concentrations (e.g. 10-25μM) from L4 stage is a convenient means to block progeny production, and has little effect on lifespan [37]. 50μM FUdR, but not lower FUdR concentrations, was sufficient to block formation of uterine tumors (Figure 5C). We then compared effects of 50μM FUdR on lifespan in wild type and *mdl-1* worms, and saw no effect on lifespan in either case (Figure 5D; Table S2). These results show that accelerated formation of uterine tumors do not cause the shorter lifespan of *mdl-1* worms. They also demonstrate that uterine tumors do not limit lifespan in wild type worms under standard culture conditions. This contrasts with the case of *daf-16* over-expression, where life shortening is suppressed by blocking germline hyperplasia either with *glp-1* or FUdR [32].

A number of interventions that remove the hermaphrodite germline cause increased lifespan, including raising *glp-4(bn2)* mutants at 25°C, and this effect is *daf-16* dependent [38, 39]. Notably, *mdl-1(0)* also reduced the longevity of *glp-4* mutants raised at 25°C (Figure 5E, Table S2). *mdl-1* shortened lifespan more in a *glp-4* background than in a wild-type background (Table S2). This suggests that *mdl-1(+)* contributes to *glp-4* longevity as well as *daf-2* longevity.

### Evidence that *mdl-1* can act in the intestine to promote longevity

**Figure 6:**
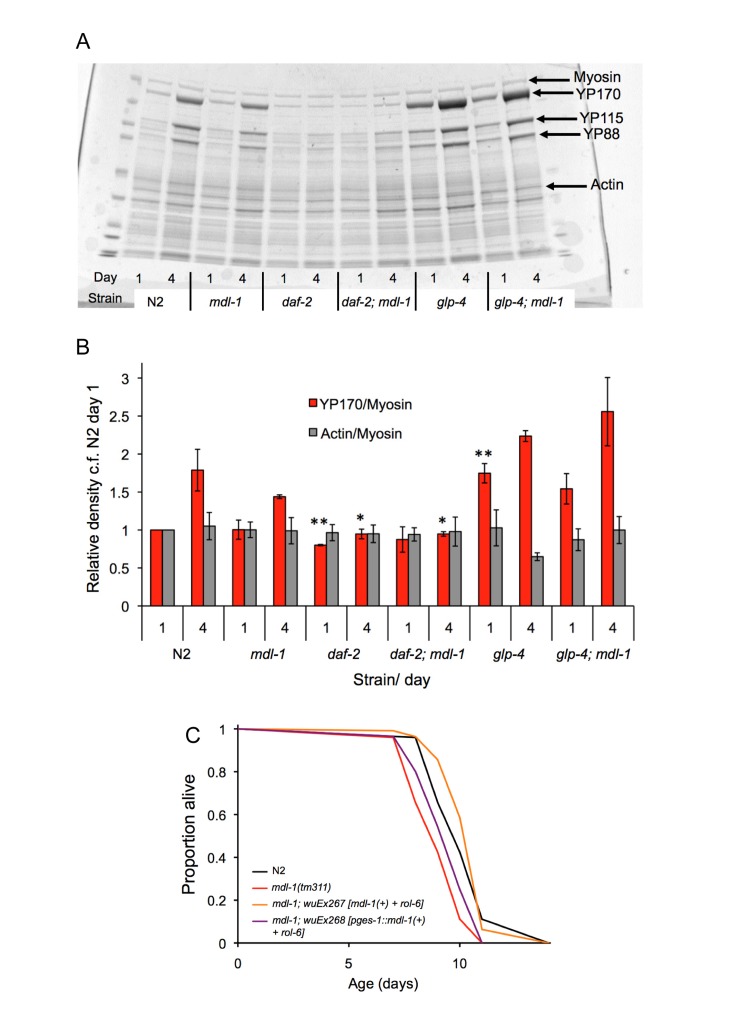
Tests for action of *mdl-1* in the intestine **(A,B)** Effect of *mdl-1* on yolk accumulation. **(A)** Example of Coomassie stained gel with *C. elegans* protein extracts. **(B)** Bar graph data is derived from densitometric measurement of protein on gels (means of 3 biological replicates; error bars, standard error). It shows levels of the major yolk protein YP170 normalized to myosin levels (which are not expected to change), and again to levels in N2 on day 1. Actin/myosin ratio gives an indication of the reliability of myosin as a standard. * 0.01 < *p* < 0.05; ** 0.001 < *p* < 0.01, compared to N2 of the same age. **(C)** Effects on lifespan of *pges-1::mdl-1(+)* rescue of *mdl-1(0)* (for statistics, see Table S3, trial 2).

The intestine plays an important role in *daf-2* mutant longevity [40], and is a site of *mdl-1* expression [10]. One possibility is that *mdl-1* affects intestinal protein synthesis. Mutation of *daf-2* causes a global reduction in protein synthesis, which may contribute to longevity [41, 42]. In aging hermaphrodites, yolk proteins (vitellogenins) become very abundant indeed [43, 44], and this accumulation is suppressed *daf-2*, apparently by inhibition of protein translation in the intestine [45] where yolk is synthesized [46]. Thus, vitellogenin accumulation rate gives some indication of intestinal protein synthesis rate. However, *mdl-1(0)* did not alter vitellogenin accumulation, either in wild type or *daf-2* mutant backgrounds (Figure 6A,B). One possibility is that *mdl-1* mutants do synthesize more vitellogenin, but due to increased laying of unfertilized oocytes, this does not result in increased vitellogenin accumulation. To check this we compared sterile *glp-4* and *glp-4; mdl-1* worms (shifted at L4 to 25°C), but again no effect of *mdl-1* was seen (Figure 6A,B).

Next, we asked whether intestine-limited rescue of *mdl-1* using the *ges-1* promoter[47] would rescue *mdl-1* shortevity. A transgene array from which *mdl-1* was expressed using its own promoter was able to restore wild-type lifespan to *mdl-1(tm311)* mutants (Figure 6C; Table S3). Notably, *pges-1::mdl-1* too increased lifespan in *mdl-1* mutants, though the effect was smaller such that lifespan was not restored to wild type. This suggests short lifespan in *mdl-1* mutants is caused by loss of *mdl-1* from several sites, including the intestine. Possibly, a second site of action of *mdl-1* on lifespan is the germline, given its impact on that tissue.

### The extent of loss of Myc among nematodes

**Figure 7:**
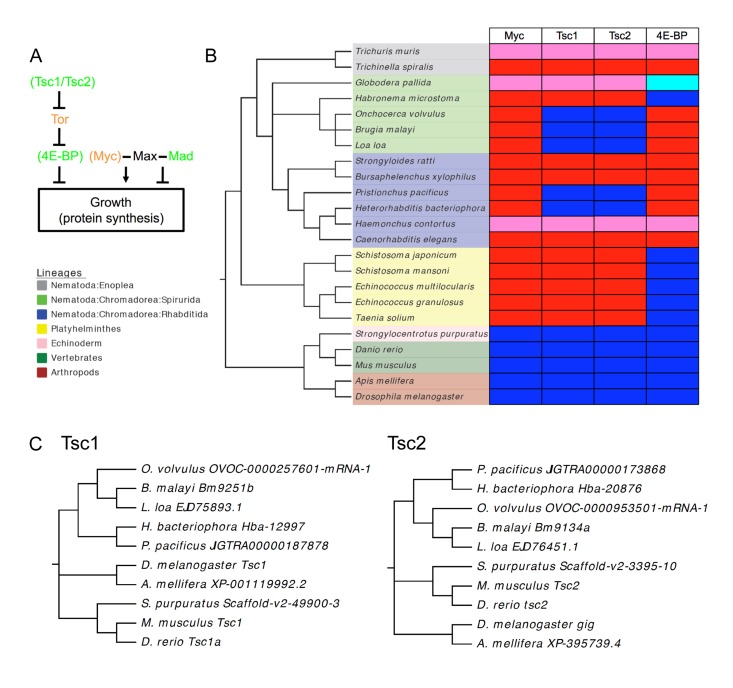
Extent of loss of Myc, Tsc1/Tsc2 and 4E-BP among the Nematoda **(A)** Outline of missing elements of Tor and Myc/Max/Mad pathways in *C. elegans*. Green, tumor-suppressor/anti-aging; orange, oncogene/pro-aging. In brackets, proteins missing from *C. elegans*. **(B)** Presence and absence of Myc, Tsc1, Tsc2 and 4E-BP among nematodes and platyhelminths. Red, absent; dark blue, present; pink, not found; pale blue, putative but somewhat divergent 4E-BP. Not found: a caveat to this analysis may be the incompleteness of the genomic sequences of some of these species with draft assemblies being available for *G. pallida* and *H. contortus* and only contigs available for *T.muris*. **(C)** Phylodendrograms of Tsc1 and Tsc2 sequences for nematodes and other animal groups. Note that species and sequence phylogenies correspond.

One puzzle relating to *mdl-1* function is the absence of Myc in *C. elegans*. In mammals, the Mad/Max/Myc system works in concert with the Tor pathways to control growth [48]. *C. elegans* also lacks key components of the Tor pathway, including the Tsc1/Tsc2 complex [49], and 4E-BP [50] (Figure 7A). To try to understand the significance of the absence of Myc in the broader context of nematode gene loss, we tested for the presence of Myc, Tsc1, Tsc2 and 4E-BP throughout the Nematoda. It was previously noted that Myc orthologs are absent not only from *C. elegans* and *C. briggsae*, but also the filarial parasite *Brugia malayi* (nematode order Spirurida)*,* and even the bilharzia parasite *Schistosoma mansoni* (phylum Platyhelminth) [51]. Searching the genomes of 13 nematode species, including representatives of the major nematode orders, no Myc orthologs were detected (Figure 7B). Myc was also absent from all 5 platyhelminth species examined. This implies that Myc evolved in the common ancestor of arthropods and chordates after divergence from the common ancestor of nematodes and platyhelminths, as previously suggested [51]. Thus, the Myc-less Mad-Max circuit in *C. elegans* appears to represent a more ancient regulatory system.

By contrast, both Tsc1/Tsc2 and 4E-BP were found in several nematode groups but were absent from many others, in a pattern indicating that each gene has been lost several times during nematode evolution. For Tsc1 and Tsc2, gene loss was correlated, i.e. both genes were either present or absent. There was no correlation between loss of Tsc/Tsc2 and 4E-BP: nematode species exist with Tsc1/Tsc2 but lacking 4E-BP and vice versa. The distribution of Tsc1/Tsc2 is surprising in that most nematode and all platyhelminth groups lack this complex, apart from spirurid nematodes, and two rhabditid species. Sequence comparisons of Tsc1 and Tsc2 protein sequences from nematodes and other animal groups is consistent with multiple instances of gene loss (rather than horizontal gene transfer) (Figure 7C).

## DISCUSSION

In this study we have shown that MDL-1 acts as an inhibitor of germline proliferation, and of oocyte hypertrophy, thereby inhibiting a salient aging-related pathology (uterine tumors). These properties of *mdl-1* mutants recapitulate effects of Mad TFs in mammals, e.g. mice lacking the Mad TF Mxi1 show hyperplasia in a number of tissues (e.g. prostatic epithelium), and are tumor prone [52]. We also confirm that MDL-1 inhibits aging, consistent with the observed association between tumor suppressors and inhibition of aging [53, 54].

### Does *mdl-1* act downstream of *daf-16*?

Our initial hypothesis was that activation of *mdl-1* expression by DAF-16 contributes to *daf-2* longevity. The results of analysis of *mdl-1* expression (Figure 3A,B) and the effects on lifespan of *mdl-1(tm311)* (Figure 4A) are consistent with this hypothesis. We also noted that *mdl-1* and *daf-2* have opposing effects on the germline: *mdl-1(0)* causes Uno-o, increased oocyte stacking and increased uterine tumors (this study), while mutation of *daf-2* has opposite effects [22, 29]. This suggested that the effects of *daf-2* on the germline might be mediated by *mdl-1*. However, epistasis analysis shows that this is not the case for oocyte production (Figure 3C). Instead *mdl-1* appears to have no effect on the germline in *daf-2* mutants, but to inhibit promotion of germline proliferation by DAF-16 in *daf-2*(+) worms (Figure 3D).

### Does *mdl-1* affect aging?

The life-shortening effect of *mdl-1(0)* is mainly the result of increased base-line hazard (Figure 4B-D). This could imply that *mdl-1(0)* does not affect aging but, rather, shortens lifespan by causing a pathology that is distinct from aging. In similar fashion, a comparison of mortality in two groups of people during the 1940s, either in Australia or interned by the Japanese, showed increased base-line hazard but not increased demographic aging in the latter [55]. It is interesting to consider whether this necessarily means that the effect of *mdl-1* on lifespan does not involve an effect on aging. The impact of *mdl-1(0)* on lifespan is greater in a *daf-2* background, suggesting that MDL-1 does contribute to *daf-2* longevity, i.e. that *mdl-1* does affect aging. Arguably, the critical point here is that something that affects demographic aging necessarily affects biological aging, while something that affects base-line hazard may or may not affect biological aging. In other words, the biological process of aging is not always the same thing as demographic aging.

One working definition of biological aging is the set of endogenously generated pathologies that increase in later life [56]. In principle, interventions that reduce one or more age-related pathology (i.e. part or all of aging) could increase lifespan by reducing base-line hazard, demographic aging, or both. One possibility is that the wider the spectrum of age-related pathologies that an intervention suppresses, the more likely will a reduction in demographic aging be seen. In conclusion, *mdl-1(0)* may or may not affect aging. Its greater effect on *daf-2* could imply that it does; however, an alternative possibility is that *mdl-1(0)* causes a pathology that is distinct from those seen during aging, and that *daf-2(-)* slightly increases the severity of this pathology, or increases its effect on mortality.

### Where and how does *mdl-1* act to impact lifespan?

The phenotype of *mdl-1(tm311)* mutants demonstrates that MDL-1 acts as a repressor of growth and proliferation in the germline. This is consistent with the role of Mad TFs as repressors of growth and proliferation in mammals [11]. A long-standing hypothesis about aging is that it is caused by molecular damage, but more recently it has been suggested that aging is caused by the run on of developmental and reproductive processes in late life, leading e.g. to pathological hyperplasia, hypertrophy and atrophy [57-59]. The action of MDL-1 as a DAF-16-activated suppressor of growth is broadly consistent with this model.

The *mdl-1* mutant phenotype also suggests that this gene may affect lifespan through its effects on the germline. One possibility is that over-production of oocytes or increased formation of uterine tumors *per se* cause a decrease in lifespan. However, our findings argue against this: suppression of these effects in *mdl-1* mutants using *glp-4* or FUdR does not suppress the life-shortening effects of *mdl-1(0)* (Figure 5B,D). A second possibility is that *mdl-1(0)* affects signaling from the germline; removal of the germline can extend lifespan, and this effect is *daf-16* dependent [39]. Consistent with this possibility, *mdl-1(0)* shortens lifespan somewhat more in long-lived germlineless *glp-4* mutants than in otherwise wild-type worms (Figure 5E).

Another possible site of *mdl-1* action on lifespan is the intestine, which plays a significant role in the control of aging [40], and where *mdl-1* is expressed [10]. Notably, intestine-limited rescue of *mdl-1(+)* in otherwise *mdl-1(0)* worms was sufficient to modestly increase lifespan. However, how *mdl-1* acts in the intestine remains unclear. Mutation of *daf-2* increases intestinal expression of *ftn-1*, which encodes the iron storage protein ferritin. This increase is wholly *daf-16* dependent and partially *mdl-1* dependent [14]. Free iron is required for growth (e.g. ferroprotein synthesis) but also generates oxidative stress. Thus, increased activity of MDL-1 in *daf-2* mutants might retard intestinal aging as part of a program of suppression of protein biosynthesis. However, we did not detect an effect of *mdl-1* on accumulation of the most abundant protein class in *C. elegans* hermaphrodites, the vitellogenins (Figure 6A,B), which are synthesized in the intestine [46]. However, it remains possible that other aspects of protein synthesis are reduced by *mdl-1*.

Alternatively, mutation of *mdl-1* might increase levels of free iron in the intestine, perhaps accelerating aging by increasing oxidative damage, as suggested by the free radical theory. Consistent with this, *mdl-1(0)* partially suppress *daf-2* Oxr (Figure 3E), suggesting that MDL-1 promotes Oxr in *daf-2* mutants. However, *mdl-1(0)* alone did not affect Oxr; moreover, free iron levels appear to have little effect on aging under standard culture conditions [60]. More broadly, a range of studies suggest that oxidative damage is not a central determinant of aging, particularly in *C. elegans* [61, 62]. Together, these findings suggest that the intestine is one of several sites of action of *mdl-1* on lifespan.

### The significance of uterine tumors in *C. elegans*

The oocyte-derived growths in the *C. elegans* uterus have been noted in previous studies and referred to as *‘tumor-like’ growths* [30], *masses* [24] and *oocyte clusters* [29]. Dictionary definitions of the word *tumor* vary, but in the common understanding of *tumor*, as in “a growth — a mass of tissue — that has no function” [63], these entities are tumors, hence our use of the term in this study. Arguably, *C. elegans* uterine tumors are both different and similar to mammalian cancer. It seems likely that *C. elegans* uterine tumors result from aging-associated overgrowth rather than mutations in oncogenes or tumor suppressor genes. But much of mammalian cancer, like worm uterine tumors, is part of the aging process. While it is clear that aging and cancer are associated, the relationship between the two remains unclear. One possibility is that aging results in changes in tissue microenvironment, e.g. due to senescent cell accumulation, that create more permissive conditions for cancer growth [64]. Aging-related tumors can occur even in the absence of transforming mutation, as in benign prostatic hyperplasia (BPH). We postulate that *C. elegans* uterine tumors, like BPH, exemplify the non-mutationally driven component of aging-associated cancer.

### The regulatory network within which *mdl-1* might function

In mammals, Mad TFs act antagonistically to Myc TFs, which promote the cell cycle, growth and apoptosis, and reduce H ferritin expression [11, 15]. Consistent with this, MDL-1 inhibits germline growth and apoptosis (this study), and activates *ftn-1* expression [14]. Moreover, in mammals over-expression of Myc can induce endomitosis and cause increased ploidy [65], often seen in tumors, and also in senescent cells [66, 67], while MDL-1 antagonizes growth of uterine tumors formed from endomitotic oocytes (this study). Thus, MDL-1 in *C. elegans* behaves as one would expect of an antagonist of Myc – which is perhaps surprising given that *C. elegans* does not possess Myc. Indeed, Myc appears to be absent from the entire Nematode phylum (this study) [51].

In mammals, the Tor pathway and Myc TFs work in concert to control protein synthesis. Myc TFs activate expression of translational machinery genes, including eIF4E, eIF4A and eIF4G, which are components of the eIF4F complex that promotes translational initiation [48]. *C. elegans* lacks many genes that are present in other animal phyla [68], which can limit its usefulness as a model organism. Besides Myc, this also affects the worm Tor pathway, which lacks several key proteins,notably Tsc1/Tsc2 and 4E-BP. Thus, *C. elegans* possesses what appears to be a different version of the IIS/Tor/Mad network of higher animals. To fully understand the worm network requires understanding these differences.

Several interpretations have been made of the absence of Myc in *C. elegans*. First, that it reflects the relatively restricted *C. elegans* cell proliferation program [10]. Second, that in *C. elegans* the Myc role is played by a different bHLH TF, for example MML-1 (Myc and Mondo-like 1) [16]. However, arguing against this interpretation, MML-1 resembles Mondo rather than Myc, it dimerizes with MXL-2 while MDL-1 dimerizes with MXL-1, and deletion of *mxl-2* has only minor phenotypic effects (abnormal migration of ray 1 precursor cells in the male tail), and does not affect e.g. growth or lifespan [16].

Another possibility is that the Myc-less Mad-Max circuit ensures rapid growth, i.e. the nematode machinery for protein translation is, in the absence of DAF-16 and MDL-1, constitutively active. Consistent with this, Tsc1/Tsc2 and 4E-BP, which both antagonize growth, are absent from *C. elegans*, and most other nematodes (Figure 7B). Notably, in *Drosophila*, inhibition of growth resulting from reduced Tor kinase activity (e.g. by over-expression of Tsc1 and Tsc2) can be rescued by overexpression of dMyc [69]. An intriguing detail is the presence of Tsc1/Tsc2 in several spirurid nematodes (Figure 7B, Table S4); notably this group includes the longest lived nematode species known, e.g. the maximum lifespan of adult *Loa loa* is at least 20 years [70]. Another possibility is that the growth inhibitory functions of Tsc1/Tsc2 and 4E-BP have been taken over by DAF-16/FoxO, which is a major regulator of protein synthesis in *C. elegans* [41, 42, 45]. Thus, perhaps DAF-16 suppresses growth in soma and germline, while MDL-1 suppresses growth in the germline alone.

### Conclusions

These results confirm that the Mad TF MDL-1 contributes to the *daf-2* longevity phenotype, and reveal a major role in inhibition of germline growth and reduction of uterine tumor development. They also suggest a role for intestinal MDL-1 in longevity assurance. The action of *mdl-1* as a DAF-16 activated gene that inhibits growth is broadly consistent with the possibility that the effects of insulin/IGF-1 signaling and DAF-16 on aging are a function of their effects on growth.

## EXPERIMENTAL PROCEDURES

### *C. elegans* culture and strains

Worms were cultured as previously described [71], at 20°C unless otherwise stated. Nematode strains used include N2 (wild type), DR1567 *daf-2(m577)III*, SS104 *glp-4(bn2)I*, GA1200 *mdl-1(tm311)X* (6X out-crossed), GA91 *daf-16(mgDf50)I; daf-2(m577),* GA1204 *daf-2(m577); mdl-1(tm311),* GA1208 *daf-16(mgDf50); daf-2(m577); mdl-1(tm311)*, GA1226 *daf-16(mgDf50); mdl-1(tm311)*, GA1230 *glp-4(bn2); mdl-1(tm311)*,GA1604 *mdl-1(tm311); wuEx267[mdl-1 + rol-6(su1006)]*, GA1605 *mdl-1(tm311); wuEx268[pges-1::mdl-1 + rol-6(su1006)]*. Primers to identify *mdl-1(tm311)* were atggaacagcaactcaaccttgg and ttaaacttggaggttgattggcaag, and heterozygotes aatgatgtgatctcgggctcg. Primers to genotype *daf-16(mgDf50)* were as described [72].

### Strain construction

Multiple mutant strains were generated using standard genetic and molecular methodologies. Strains carrying mutations on the X chromosome (e.g. *mdl-1(tm311)*) were crossed with N2 males to generate hemizygous mutant males which were mated with L4 hermaphrodites of the strain carrying the second mutation of interest. F1 offspring were picked, and allowed to self-fertilise and 80 F2 were picked, allowed to lay eggs overnight, lysed and stored at −20°C. Genomic deletions were identified using PCR. In the presence of the temperature sensitive *daf-2(m577)* allele, F1 animals were shifted to 25°C to select for dauer formation in the F2, dauers were picked and left to recover at 15°C to lay eggs, the F2 were lysed and tested for deletions. The *daf-16(mgDf50); daf-2(m577); mdl-1(tm311)* triple mutant was constructed by mating *daf-16; daf-2* males with *daf-2; mdl-1* hermaphrodites. The F2 generation was cloned, lysed and offspring raised at 25°C. *daf-16(mgDf50)* homozygotes in the F3 were initially identified as non-dauer formers, and *mdl-1(tm311)* homozygotes identified by PCR.

*mdl-1* transgenic lines were created by microinjection using PCR products and PCR fusion [73]. Primers used to make GA1604 were aaattgcacatgcagagacg and gaaagatacggaaggtgtgc. Primers used to make GA1605 were ttgtctattggtatggctgc; ggttgagttgctgttccattacaaggaa tatccgcatctg; gcgctaccaataaggctaag; aatggaacagcaactc aacc; gaaagatacggaaggtgtgc; and tttacaacacgatccacacg.

### Staining protocols

To quantify germ cell number, nuclei were stained using DNA-binding dye 4',6-diamidino-2-phenylindole (DAPI). Animals were fixed in methanol, washed with M9 buffer and incubated in the dark in a 500ng/μl DAPI solution for 30 min. Thereafter they were washed again in M9 buffer. To quantify the number of apoptotic cells in living animals, nematodes were stained with SYTO 12 Green Fluorescent Nucleic Acid Stain (Molecular Probes). The animals were incubated in the dark in a 33μM SYTO 12 solution for 4 hr, and then placed on an OP50 lawn for 1 hr.

### Quantitative RT-PCR and chromatin immunoprecipitation PCR (ChIP-PCR)

RT-PCR and ChIP-PCR were performed largely as previously described [9, 74]. Primers for RT-PCR amplification from *mdl-1* mRNA were cccgtttgcgtgtcattgt and atggattgtgagagtgttgagaat. Primers for ChIP-PCR of an *mdl-1* promoter region (Figure 1A) were ccccctcgttttctccatgt and gccgctcgctccaatg.

### Microscopy

Freshly prepared agar pads were created by dropping 35μl of 2% agarose onto a glass slide. Worms were anaesthesised using 5μl 0.2% levamisole. Nomarski microscopy was performed on a Zeiss Axioskop2 plus microscope with a Hamamatsu ORCAER digital camera C4742-95. Images were acquired using Volocity 5.5 software, with 10x eyepieces and a 40x objective lens. For body size measurements, worms were synchronized and hatched overnight in M9 buffer. The next day, ~30 worms per strain were imaged, and the remainder cultured on OP50 and thereafter imaged consecutively for 6 days. Volocity 5.5 was used to quantify the length of the worm from head to tail and the width across the pharyngeal-intestinal valve region.

### Uterine tumor scoring system

Uterine status was scored from 1 to 5, where scores of 3-5 indicates the presence of a tumor. Class 1 denotes a normal, youthful uterus containing fertilized eggs and/or unfertilized oocytes of normal appearance. Class 2 denotes a uterus whose contents appear somewhat abnormal, but without a clear increase in size. Class 3 denotes a uterus containing a small tumor. Class 4 denotes a uterus containing a medium sized tumor, that does not fill the body cavity in the mid-body region. Class 5 denotes a uterus where the tumor is large, and fill the entire body cavity in the mid-body region, and even causes distension of the body wall. For the scoring, the tumor images was randomised, and the scoring was performed blinded by 3 different scorers. The non-parametric Wilcoxon test was used to compare tumor classes between worm strains and within the same strain on day 1 and day 4. The analysis was performed using the statistical programme JMP 9 (SAS Institute Inc.)

### Fertility measurements

Brood sizes were assayed as previously described [22]. Briefly, 10-12 L4 hermaphrodites, raised at 20°C were cloned on individual plates, shifted to 25°C and transferred daily for 7 days. Plates were incubated at 20°C for 24 hr to allow offspring to hatch and then larvae, unfertilized oocytes and dead eggs were scored.

### Yolk level measurements

For each test sample, 50 hermaphrodites at day 1 and 4 of adulthood were transferred into an Eppendorf tube filled with 1 ml M9 buffer. Worms were spun at 800 rpm for 2 min and supernatant removed, leaving 25 μl. Then 25 μl of 2x Laemmli buffer (Sigma) was added, and samples incubated at 70°C for 15 min, vortexed every other minute and then shifted to 95°C for 5 min. Lysates were centrifuged at 13,000 rpm at 4°C for 15 min. 20 μl of each sample was loaded onto a Criterion XT Tris-Acetate gel. The gel was run at 200V in SDS-PAGE chamber with 1x 3-(N-morpholino) propanesulfonic acid (MOPS) buffer for 45 min. The gel was fixed in methanol, acetic acid and ultrapure water in ratio of 50:10:40 for 30 min. The fixing solution was then discarded and replaced with Coomassie fixation solution (50:3:40:10 methanol: Coomassie stock solution: ultrapure water: acetic acid). 12.0 g of Brilliant Blue R-250, 300 ml methanol and 60ml acetic acid were used to prepare Coomassie stock solution. The gel was incubated overnight in destaining solution (45:10:45 methanol: acetic acid: ultrapure water). Protein bands on the gel were visualised by a Image Quant GE Healthcare scanner system connected to a computer, and analysed by ImageQuant software with which densitometry was performed. Each experiment was done in triplicate.

### Lifespan measurements

These were conducted as previously described [22]. Briefly, 5 plates per condition were seeded with OP50 2 days before the start of the experiment. 10 μM FUdR was topically applied before beginning the trial. Animals were raised at 20°C, or for assays including *glp-4(bn2)* mutants, animals were raised at 15°C and switched to 25°C at L4 stage. All animals were transferred to fresh plates on day 5 and 10. Deaths were scored and losses due to causes other than death were censored. Lifespan data were deposited in SurvCurv [75] <IDs filled in proof>.

### Dauer formation measurement

Dauer formation was assessed at 22.9°C as previously described [22]. Briefly, 12 L4s were picked and raised to adulthood at 20°C for 2 days. These gravid adults were then placed on 35mm plates to lay eggs for 6 hrs at the test temperature, after which the adults were removed and the larvae were allowed to develop at the test temperature. Dauers and normal larvae were scored 72 hr after the midpoint of the egg lay and the percentage dauer formation was calculated by dividing the number of dauer larvae by the total number of offspring.

### Oxidative stress resistance

1 day old adults were tested for resistance to 7.5 mM *tert*-butylhydroperoxide (*t*-BOOH) as previously described [33]. Briefly, L4 animals were picked from mixed stage plates raised at 20°C, then shifted to 25°C overnight. NGM agar was supplemented with 7.5 mM *t*-BOOH and the plates were left to dry overnight. The next day, each plate was supplemented with a blob of densely grown OP50, and 15 young adults were added per plate. The trial was conducted at 25°C and animals were scored every 2 to 3 hr until the last animal had perished.

### Bioinformatics

Myc, Tsc1, Tsc2 and 4E-BP orthologs were sought by local alignment searches of the 4 protein sequences to the gene models in *Mus musculus* using BLASTP searches. Since not all genomes were available in WormBase, orthologs for the parasitic helminths (*B. xylophilus*, *E. granulosus, E. multilocularis, T. solium, H. microstoma*, *S. mansoni* and *S. japonicum*) were derived from GeneDB. Orthologs for *G. pallida, H. contortus, O. volvulus, S. ratti and T. muris* were sought using the data available on the Sanger Institute Resources. Orthologs to *T. muris* were sought by local alignment searches of the 4 genes to contigs, using a “protein versus translated DNA” a TBLASTN search. Finally, orthologs of *P. pacificus* were sought among the gene predictions available from www.pristionchus.org. Multiple sequence alignments of the Tsc1/2 protein sequences were done using MUSCLE [76]. All trees were constructed and visualised as previously described [74].

### Statistical analysis

Lifespans were analysed using the Cox Proportional Hazard method with the Efron approximation for ties of the survival package in R. The logistic mortality models were fitted to the lifespan data and parameter difference tested using the Survomatic R package. The body sizes were analysed using a linear regression model taking into account the trial as random factor in R. The Wilcoxon Mann test was used for tumors and the brood sizes were compared using a standard Student's t Test.

## SUPPLEMENTAL TABLES


