# Supplementary material for: MDL-1, a growth- and tumor-suppressor, slows aging and prevents germline hyperplasia and hypertrophy in C. elegans
Source: Aging (Albany NY). 2014 Feb 16;6(2):98–117. doi: 10.18632/aging.100638 (PMC3969279; doi:10.18632/aging.100638)
Supplement: Supplementary file 1 [file aging-06-098-s001.pdf]

mediate the effects of insulin/IGF-1 signaling on aging in C. elegans. PLoS Genet. 2013; In press.
75. Ziehm M and Thornton JM. Unlocking the potential of survival data for model organisms through a new database and online analysis platform: SurvCurv. Aging Cell. 2013; 12:910-916.
76. Edgar RC. MUSCLE: a multiple sequence alignment method with reduced time and space complexity. BMC Bioinformatics. 2004; 5:113.

SUPPLEMENTAL TABLES

Table S1. Effect of *mdl-1* on life span in *C. elegans.* The trials were performed at 25°C without FUdR. Genotype: N2 wildtype, GA1200 *mdl-1(tm311),* DR1567 *daf-2 (m577),* GA1204 *daf-2(m577); mdl-1(tm311)*. *p*, log rank test. [n] biological replicates number. [C], combined data from 4 trials.

Genotype	Deaths/ censored	Mean life span (days)	% *vs.* wild type	*p vs.* wild type	% *vs. daf-2*	*p vs. daf-2*
Wild type	**[C] 513/18**	**14.7**				
	[1] 120/5	15.2				
	[2] 121/5	14.1				
	[3] 133/7	15.3				
	[4] 139/1	14.3				
mdl-1(tm311)	**[C] 500/17**	**12.8**	**-13.1**	**<.0001**		
	[1] 119/4	14.4	-5.4	0.0288		
	[2] 115/4	12.0	-15.1	<.0001		
	[3] 125/8	12.9	-16.0	<.0001		
	[4] 141/1	12.1	-15.5	<.0001		
daf-2 (m577)	**[C] 412/57**	**35.2**	**+139.2**	**<.0001**		
	[1] 87/26	31.0	+104.4	<.0001		
	[2] 86/3	34.0	+141.1	<.0001		
	[3] 121/8	35.1	+129.2	<.0001		
	[4] 118/20	39.2	+174.1	<.0001		
daf-2(m577);	**[C] 438/78**	**26.6**	**+80.9**	**<.0001**	**-24.5**	**<.0001**
mdl-1(tm311)	[1] 75/59	21.2	+39.4	<.0001	-31.7	<.0001
	[2] 113/1	28.0	+98.5	<.0001	-17.6	<.0001
	[3] 132/4	27.0	+76.4	<.0001	-22.9	<.0001
	[4] 118/14	28.3	+97.9	<.0001	-27.9	<.0001

Table S2. ***glp-4(bn2)*** **and FUdR do not suppress** ***mdl-1*** **shortevity.** Genotypes: N2 wildtype, GA1200 *mdl-1(tm311),* SS104 *glp-4(bn2)*, GA1230 *glp-4*; *mdl-1*. p, log rank test. [n] biological replicates number. *glp-4* trial, worms raised at 15˚C to L4, then shifted to 25˚C.

Genotype	Deaths/ censored	Mean life span (days)	% *vs.* wild type	*p vs.* wild type	% *vs. glp-4*	*p vs. glp-4*
glp-4 **Trials** (worms raised at 15˚C, shifted at L4 stage to 25˚C)						
Wild type	[1] 77/11	15.5				
	[2] 69/11	13.2				
mdl-1(tm311)	[1] 61/11	12.9	-17.0	0.0002		
	[2] 48/11	11.3	-14.5	0.0023		
glp-4(bn2)	[1] 86/14	15.7	+1.59	0.46		
	[2] 64/9	14.3	+8.4	0.43		
glp-4; mdl-1	[1] 120/4	12.3	-4.4	<0.0001	-21.6	<0.0001
	[2] 75/6	9.1	-19.2	<0.0001	-36.3	<0.0001
glp-4 **Trials** (worms raised and maintained at 25˚C)						
Wild type	[1] 87/7	12.2				
	[2] 78/12	14.2				
mdl-1(tm311)	[1] 78/19	11.4	-6.6	0.20		
	[2] 76/22	12.7	-10.7	0.05		
glp-4(bn2)	[1] 94/9	14.0	+14.8	0.0035		
	[2] 77/16	17.6	+24.0	0.0002		
glp-4; mdl-1	[1] 83/17	12.3	+0.8	0.21	-12.0	0.024
	[2] 70/17	15.0	-15.2	0.36	-14.7	0.0051
**FUdR Trials**			**% *vs.* wild type (no FUdR)**	***p vs.* wild type (no FUdR)**	**% *vs.* no FUdR**	***p vs.* no FUdR**
Wild type	[1] 46/14	17.5				
	[2] 41/19	16.8				
mdl-1(tm311)	[1] 49/11	12.4	-41.1	<0.0001		
	[2] 49/11	13.3	-26.3	0.0027		
Wild type (50µM FUdR)	[1] 55/5	17.3	-1.2	0.55	-1.2	0.55
	[2] 57/3	14.6	-15.1	0.022	-15.1	0.022
mdl-1(tm311) (50µM FUdR)	[1] 54/6	12.7	-37.8	<0.0001	+2.4	0.80
	[2] 49/11	11.6	-44.8	<0.0001	-14.7	0.032

Table S1. Effect of *mdl-1* on life span in *C. elegans.*

Strain	Deaths/ censored	Mean life span (days)	% *vs.* wild type	*p vs.* wild type	% *vs. mdl-1*	*p vs. mdl-1*
Wild type	**[C] 198/0**	**11.30**				
	[1] 99/0	11.83				
	[2] 99/0	10.97				
mdl-1(tm311)	**[C] 198/1**	**9.36**	**-17.97**	**<0.0001**		
	[1] 99/0	9.57	-19.10	<0.0001		
	[2] 99/1	9.15	-15.20	<0.0001		
mdl-1; wuEx267	**[C] 191/0**	**10.91**	**-3.45**	**0.0060**	**+19.55**	**<0.0001**
[mdl-1 rol-6]	[1] 80/0	11.36	-3.97	0.04	+18.70	<0.0001
	[2] 111/0	10.59	-1.85	0.22	+15.74	<0.0001
mdl-1; wuEx268	**[C] 210/0**	**9.97**	**-11.77**	**<0.0001**	**+9.51**	**<0.0001**
[pges-1::mdl-1	[1] 94/0	10.47	-11.50	<0.0001	+9.40	<0.0001
rol-6].	[2] 116/0	9.56	-11.40	<0.0001	+4.48	0.0059
